# Whole-genome scanning for the litter size trait associated genes and SNPs under selection in dairy goat (*Capra hircus*)

**DOI:** 10.1038/srep38096

**Published:** 2016-12-01

**Authors:** Fang-Nong Lai, Hong-Li Zhai, Ming Cheng, Jun-Yu Ma, Shun-Feng Cheng, Wei Ge, Guo-Liang Zhang, Jun-Jie Wang, Rui-Qian Zhang, Xue Wang, Ling-Jiang Min, Jiu-Zhou Song, Wei Shen

**Affiliations:** 1 Key Laboratory of Animal Reproduction and Germplasm Enhancement in Universities of Shandong, College of Animal Science and Technology, Qingdao Agricultural University, Qingdao 266109, China; 2 Shandong International Biotechnology Park, Yantai 264670, China; 3 Qingdao Research Institute of Husbandry and Veterinary, Qingdao 266300, China; 4 College of Life Sciences, Qingdao Agricultural University, Qingdao 266109, China; 5 Department of Animal and Avian Sciences, University of Maryland, College Park, Maryland 20742, USA

## Abstract

Dairy goats are one of the most utilized domesticated animals in China. Here, we selected extreme populations based on differential fecundity in two Laoshan dairy goat populations. Utilizing deep sequencing we have generated 68.7 and 57.8 giga base of sequencing data, and identified 12,458,711 and 12,423,128 SNPs in the low fecundity and high fecundity groups, respectively. Following selective sweep analyses, a number of loci and candidate genes in the two populations were scanned independently. The reproduction related genes *CCNB2, AR, ADCY1, DNMT3B, SMAD2, AMHR2, ERBB2, FGFR1, MAP3K12* and *THEM4* were specifically selected in the high fecundity group whereas *KDM6A*, *TENM1*, *SWI5* and *CYM* were specifically selected in the low fecundity group. A sub-set of genes including *SYCP2*, *SOX5* and *POU3F4* were localized both in the high and low fecundity selection windows, suggesting that these particular genes experienced strong selection with lower genetic diversity. From the genome data, the rare nonsense mutations may not contribute to fecundity, whereas nonsynonymous SNPs likely play a predominant role. The nonsynonymous exonic SNPs in *SETDB2* and *CDH26* which were co-localized in the selected region may take part in fecundity traits. These observations bring us a new insights into the genetic variation influencing fecundity traits within dairy goats.

The goat (*Capra hircus*) is one of the oldest domesticated animal species^1^. From the statistical data available from the UN Food and Agricultural Organization in 2011, there are more than 1000 breeds of goats and 924 million live goats around the world^2^. Domesticated goats are generally used for producing milk, meat, fiber and hides^1^. Among the domesticated breeds dairy goats are considered particularly useful from a production standpoint. A study by Olivier *et al*. reported that the population of dairy goats accounts for 19.1% of the total goat numbers present in developing countries^3^. The number of dairy goats in 2008 totaled nearly 5.8 million in China, located mainly in Shaanxi, Shandong, and Henan provinces^4^. Several key dairy goat breeds live in China, including Guanzhong dairy goats, Xinong Saanen dairy goats, and Laoshan dairy goats. However, the dairy goat industry is still not meeting consumer demand for milk and therefore there is an urgent need to further improve dairy goats’ reproduction and production traits.

Litter size (LS) is a critical and complicated economic trait within the goat industry. Litter size appears to be controlled by multiple genes and factors^5^ including, ovarian follicular development, oocyte maturation, ovulation, fertilization, embryogenesis, embryo implantation, and uterine receptivity. These fecundity traits are regulated by gonadotropins, ovarian steroid hormones, and growth factors including luteinzing hormone (LH), follicle stimulating hormone (FSH), 17β-estradiol (E2), progesterone (Prog), and activin A (a protein complex belong to the TGF-β protein superfamily). Inactive homozygous mutations occurring in transforming growth factor β (TGFB) superfamily members including BMP15 or GDF9 results in decreased ovulation rates leading to eventual sterility^6^,^7^.

Many genetic markers have been associated with goat litter size. A previous study demonstrated that a non-synonymous SNP G1534A mutation in exon 2 of bone morphogenetic protein 4 (BMP4) within the India goat breeds Black-Bengal and Jakhrana are related to variations seen in prolificacy^8^. Similarly, two homozygous SNPs (g.151435C > T, g.173057T > C) located in exon 2 and the 3′ untranslated region (3′ UTR) of the prolactin receptor (PRLR) gene are significantly associated with fecundity in both Guanzhong and Boer goat breeds^9^. Furthermore, it has been found that heterozygous SNPs g.224 A > G and g.227 C > T in the 5′ flanking region in insulin-like growth factor 1 (IGF1) is associated with LS^10^.

The first domestic goat *de novo* genomes were sequenced and assembled in 2013 and since that time it has been feasible to screen for domestic traits^11^. However, it is still critical to select excellent individuals based on the genetic features of interest.

Therefore, in this study we adopted modern next generation sequencing techniques in order to screen for high fecundity linked single nucleotide polymorphisms (SNPs). In the present study we focused on Laoshan dairy goats^4^. They have the characteristics of seasonal estrus, milk yields reaching up to 800 kg in 270 days, litter sizes averaging 1.7 kids, and are mainly distributed in the eastern part of Shandong Province in China^4^,^12^. We selected extreme populations in order to help disseminate the genetic differences. High and low fecundity ewes of Laoshan dairy goats were analyzed separately. DNA from each ewe was extracted and pooled equally based on their fecundity grouping. We performed high throughput sequencing and sweeping analysis of each group based on genome wide SNP comparisons.

## Materials and Methods

### Ethics Statement

All procedures conducted in this study were observed by the administration of animal affairs and approved by the Ethics Committee at Qingdao Agricultural University, and all procedures were in accordance with the agreement of Ethics Committee at Qingdao Agricultural University (Agreement No. 2013–16).

### Animals and whole genome sequencing of pools

In the present study, we compared two groups of Laoshan dairy goats with differing reproductive properties. The goats were housed at Aote goats’ farm in Qingdao, Shandong province, China.

Genomic DNA was extracted from the ear tissue of each goat using the QIAamp DNA Mini Kit (QIAGEN, 51304, Hilden, Germany). The low yield group consisted of 20 individuals each with a litter size of one. The high yield group consisted of 14 individuals, with 13 having a litter size of 3 and 1 have a litter size of 4. DNA from each group member was pooled in equimolar quantities (2 μg/sample) in order to establish the two pair-end sequencing libraries (insert sizes approximate ~0.5 kb, with the effective insert sequencing concentration >2 nM). The sequencing libraries construction and sequencing were performed on an Illumina HiSeq 2000^TM^ platform by Beijing Novogene Bioinformatics Technology Co., Ltd.

### Sequence quality checking and mapping

Before alignment, we initially filtered out the adapter and low quality raw paired reads as described in Li *et al*.^13^, with minor modifications as follows: (a) reads with >50% bases having a Q_phred_ score < = 5; (b) reads with > = 10% unidentified nucleotides (N); (c) reads with >10 nt aligned to the adapter sequence. Secondly, we utilized BWA software^14^ (the command line used was: ‘aln -e 10 -l 32 -i 15 -q 10’) to map the high-quality clean reads to the goat reference genome sequence (CHIR_1.0, http://www.ncbi.nlm.nih.gov/genome/?term=Capra_hircus). The output of SAM formatted data was then cleaned using SAMtools to remove replicates^15^.

### SNP calling and annotation

After alignment, we called SNPs for the two groups using the command ‘mpileup’ in SAMtools with the parameters set as ‘-m 2 -F 0.002 -d 1000’. In order to obtain the high quality SNPs, we only included the called SNPs with reads >4, and quality > = 20 in the analysis. To identify nonsense, nonsynonymous, and synonymous candidate mutations we utilized ANNOVAR to annotate the SNPs^16^.

### Sweep analysis of genes and SNPs in selected regions

We performed selective sweep analysis by using the 2 sequenced pools. We separately performed 2 approaches based on genetic differentiation (Fst) and heterozygosity scores (Hp) of each 150 KB genome window. Fst was calculated by using PoPoolation 2^17^, and Hp was calculated by using a local Perl script. Briefly, 
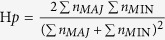
, where n_*MAJ*_ and n_*MIN*_ are the sum of major and minor allele frequencies at the given specific 150 KB window. After Z-transformation the selected windows were used by selecting top 5% in ZHp scores for each group and the Fst scores intersection. Data visualization was done by utilizing circos, IGV, geneview module of python and the ggplot package of R.

### Sanger sequencing validation

To confirm the altered translation SNPs, we designed PCR primers (Table 1). The PCR products from a random selection of 10 samples of the LF group and 10 samples from the HF group were processed using Sanger sequencing, which was conducted by the GENEWIZ Company (JiangSu, China).

### Data access

The raw sequencing data generated in this study has been uploaded to the NCBI SRA database with the accession number: PRJNA322364 (http://www.ncbi.nlm.nih.gov/bioproject/PRJNA322364/).

## Results

### Sequencing and mapping

We individually purified genomic DNA of 34 Laoshan dairy goats with significantly different fecundity (P < 0.01). We selected goats with low fecundity (LF) (n = 20, LS = 1.00 ± 0.00) and high fecundity (HF) (n = 14, LS = 3.07 ± 0.27), and pooled their genomic DNA equivalently. After library construction and sequencing with an Illumina HiSeq^TM^ 2000, 68.73 and 57.81 GB of raw data was generated (Table 2). After filtered out the low quality reads a total of 23.74 × and 25.18 × depth of LF and HF pools were mapped to the goat reference genome, with mapping rates of 98.46% and 98.11% (Tables 2 and 3).

### SNP calling and annotation

After mapping to the Yunnan black goat reference genome, we then called SNPs and annotated them in the LF and HF groups. We identified a total of 12,458,711 and 12,423,128 SNPs within the LF and HF groups, respectively. The annotation and distribution of SNPs is shown in Table 4. The transition-to-transversion ratio (ts/tv) of LF and HF groups were 2.305 and 2.299, respectively. The exonic SNPs were counted, with 440 vs. 380 lead to stop gain variation, 60 vs. 62 lead to stop loss nonsense variation and 37,755 vs. 37,501 lead to nonsynonymous mutations for the LF and HF groups.

**Figure 1:**
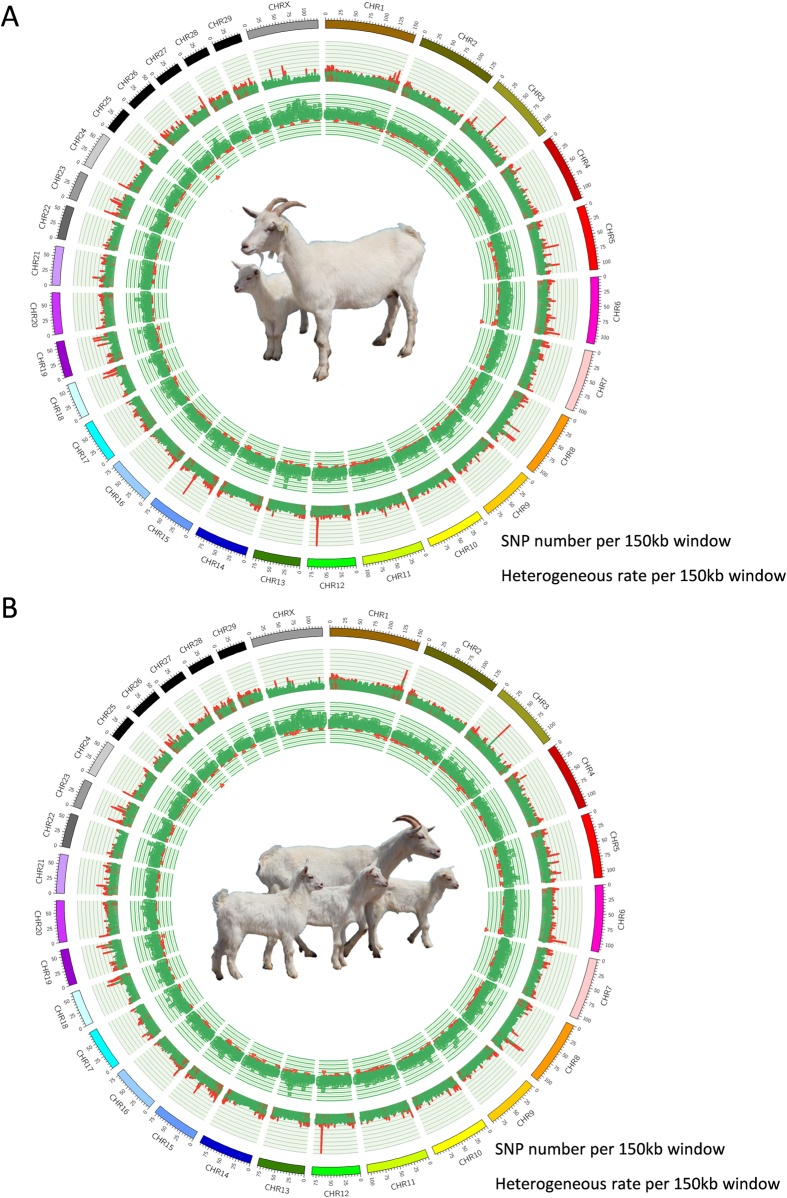
Circos plot of SNP distribution in goat chromosomes. SNP distribution in (**A**) the low fecundity (LF) pooled group and (**B**) the high fecundity (HF) pooled group. The outermost circle depicts the ideograms of each chromosome. The rules indicate the length and position of each chromosome. The middle circle shows the called SNP number per 150 KB genome window, the range between 0–4000, green line shows SNP numbers < = 1500 per the window, whereas a red line represents SNP numbers >1500 within the window. The internal circle shows the SNPs average heterozygosity per window, with a range between 0–100%. The green or red triangle represents heterozygosity > = or <40%, respectively.

We counted the SNPs and averaged the SNPs heterozygocity per 150 KB read windows, the results are depicted in Fig. 1. Total SNP number per each 150 KB read window was similar between the LF and HF groups (Fig. 1). However, compared with the LF group the average SNP heterozygocity showed a drastic decrease in the HF group in the 67.05–67.50 and 82.35–83.10 MB regions in chromosome X, 10.65–10.80 MB region in chromosome 18, 82.35–82.50 MB region in chromosome 12, and 65.70–66.15 MB region in chromosome 8. Also, the average of SNP heterozygocity was lower in the LF group when compared to the HF group in the 68.40–68.70 MB region in chromosome 12, 84.90–85.35 MB region in chromosome 5, 115.65–115.95 MB region in chromosome X, and the 77.40–77.55 MB region in chromosome 11 (Fig. 1 and Table S1). These regions of dramatic heterozygocity differences may result from the dividing of the two groups.

### Genome-wide selective sweeping analysis

**Figure 2:**
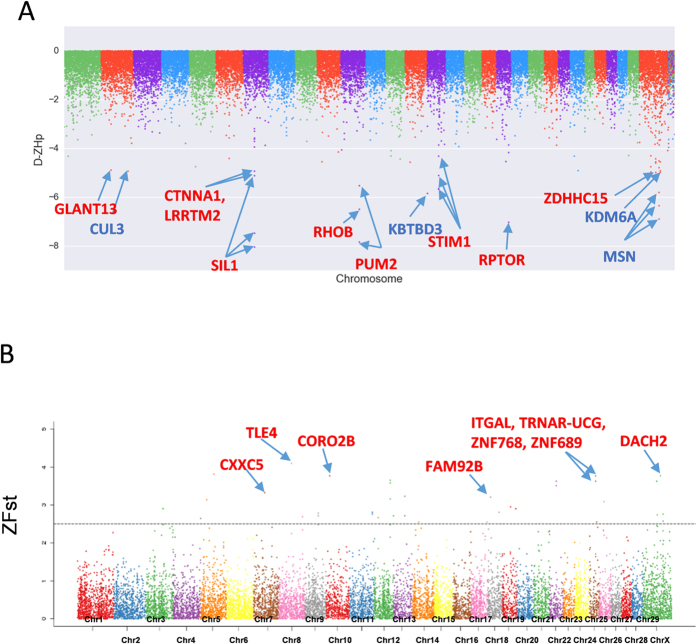
(**A**) Genome-wide Z-transformation of differential heterozygosity (D-ZHp, ZHp_-HF_- ZHp_-LF_) manhattan plot between LF and HF groups. Red names are of some of genes contained in the minimum D-ZHp group in the 150 KB window. Names of red or blue mean homozygosis in the HF or LF groups. (**B**) Genome-wide Z-transformation of F-statistics (Z-Fst) manhattan plot between LF and HF groups. Red names are of some of the genes contained in the maximum Z-Fst in the 150 KB window.

In order to screen the genomic regions we calculated the total SNP counts in each region of the genome over different lengths (50, 100, 150 and 200 KB). We found that when the size of the window is > = 150 KB the total SNPs count was less than 20 (Fig. S1). Therefore, the results suggested that 150 KB is an adequate size for the sweeping analysis. We then calculated the pooled heterozygosity (Hp) of each 150 KB window, and used an overlap of 75 KB as the sliding size. The Hp was estimated from genetic polymorphism data and used in population genetic analysis. 33,643 chromosome windows were assessed in the Z-transformation of the Hp (ZHp) scores, ranging from −11.72 to 4.36, and −12.53 to 4.24 in both the LF and HF groups. Due to the fact that LF and HF groups are from the same breed of dairy goat differing only in litter size, most ZHp scores showed little variation between the LF and HF groups. We calculated the different values of ZHp between the LF and HF groups. After the selected sweep analysis we determined the homozygous SNPs within the windows in the HF group and heterozygous in the LF group. The most representative region was 46.58–46.65 MB in chromosome 7 (D-ZHp = −8.05, LF-ZHp = 2.50, HF-ZHp = −5.55), which contains the SIL1 gene (*SIL1 nucleotide exchange factor*). The other strongly selected genomic regions were 77.40–77.47 MB (D-ZHp = −7.84) in chromosome 11, 47.70–47.85 MB (D-ZHp = −5.67) in chromosome 15, and 46.35–46.50 MB (D-ZHp = −5.12) and 68.55–68.70 MB in chromosome X. These regions contain RHOB (*ras homolog family member B*), PUM2 (*pumilio RNA-binding family member 2*), RRM1 (*ribonucleotide reductase M1*), STIM1 (*stromal interaction molecule 1*), CTNNA1 (*catenin alpha 1*), LRRTM2 (*leucine rich repeat transmembrane neuronal 2*) and ZDHHC15 (*zinc finger, DHHC-type containing 15*), respectively (Fig. 2A).

**Figure 3:**
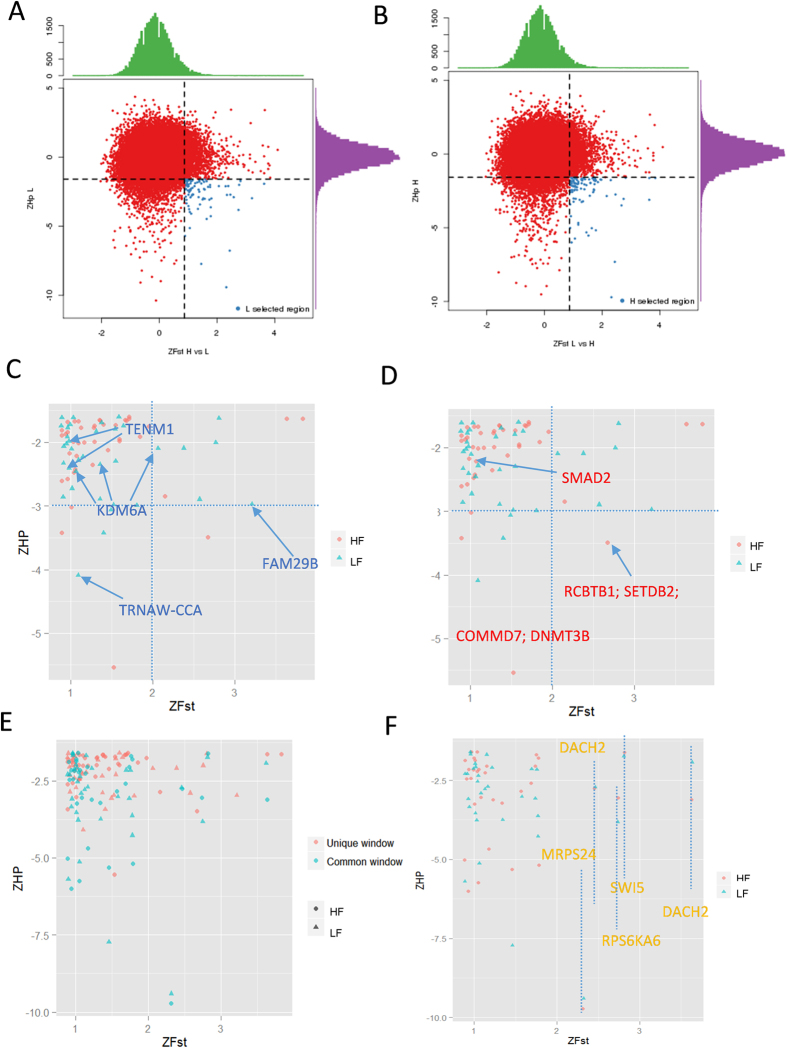
Distribution between Fst versus ZHp. (**A**) Fst versus ZHp of each window in the LF group, the blue points show candidate windows different from the HF group and with more homozygous SNPs. (**B**) Fst versus ZHp of each window in HF group, the blue points represent candidate windows different from the LF group and with more homozygous SNPs. (**C**) Distribution of unique and shared candidate windows in the LF and HF groups, blue and red names are of candidate gene uniquely localized in the LF (**E**) and HF (**F**) groups. Distribution of shared candidate windows in the LF and HF groups. (**D**) The yellow color names are of the genes contained in the candidate windows.

F-statistics (Fst) is another descriptive statistic and measure of population genetic differentiation experienced under nature or artificial selection^18^. Therefore, we calculated the Fst and Z-transformation (ZFst) as well. After measuring and sorting, the most significantly annotated differential genetic regions were scanned for genes. Region 54.15–54.90 MB in chromosome 8 contains TLE4 (*transducing-like enhance of split 4*) and is the most differential region between the LF and HF groups (Fst = 0.284, and ZFst = 4.0965). After artificial selection regions showed differentiation between the LH and HF groups, such as 15.23–15.30 MB (ZFst = 3.769) in chromosome 10, 26.70–26.85 MB (ZFst = 3.769) in chromosome 25, 26.78–26.85 MB (ZFst = 3.6286) in chromosome 25, and 58.50–58.65 MB (ZFst = 3.6286) in chromosome X, which contained CORO2B (*coronin, actin binding protein, 2B*), TRNAR-UCG (*transfer RNA arginine, anticodon UCG*), ITGAL (*integrin alpha L*), ZNF768 (*zinc finger protein 768*), ZNF689 (*zinc finger protein 689*) and DACH2 (*dachshund family transcription factor 2*) (Fig. 2B). Combined with the ZHp and ZFst of Score, we selected the 5% of the minimum ZHp score and maximum 5% ZFst score intersection window, which represent the high homology region within group and experienced dramatically differentiation result from selection and grouping in the LF (Fig. 3A blue points) and HF (Fig. 3B blue points) groups.

**Figure 4:**
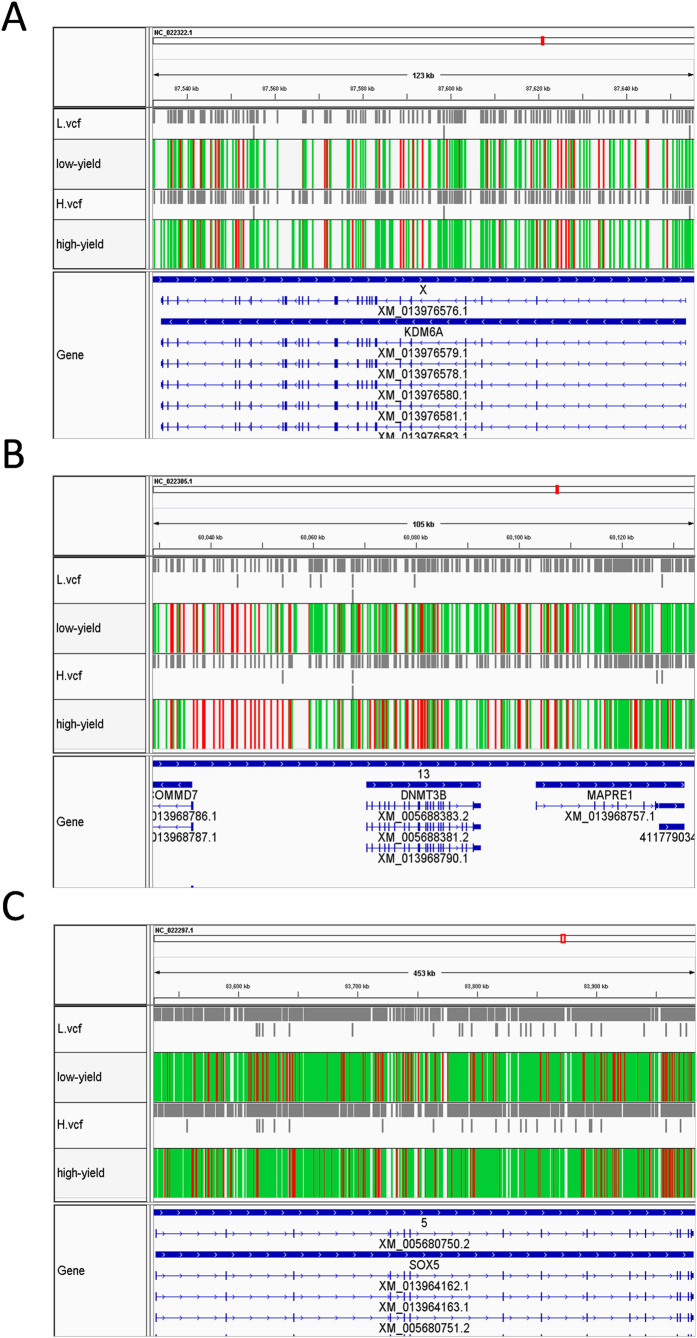
Genes in the candidate ZFst-DZHp regions. Red lines show homozygous SNPs, and green lines show heterozygous SNPs. (**A**) KDM6A localized in the LF unique candidate window, (**B**) DNMT3B in the HF unique candidate window, and (**C**) SOX5 in the shared candidate window.

Next, we searched the selected region with the highest differentiation (top 5% Fst scores) and highest homozygosity (bottom 5% ZHp) for each group (Fig. 3A,B). Interestingly, a set of shared overlapping windows between HF and LF groups were selected (Fig. 3C,D). This suggests genes in shared regions such as DACH2, SWI5, and MRPS24 containing SNPs are very different between the LF and HF group and also contain more homozygous SNPs. Next, we screened the unique homologous SNP containing regions in the HF and LF groups (Fig. 3E,F). Screening selected 73 fixed unique genome windows containing 137 candidate genes in the HF group (Table S2). Within the candidate HF groups a number of reproduction-related genes were selected including the oocyte meiosis related genes CCNB2 (*cyclin B2*), AR (*androgen receptor*), ADCY1 (*adenylate cyclase 1*), DNMT3B (*DNA cytosine - 5 - methyltransferase 3 beta*), SMAD2 (*SMAD family member 2*) and the AMHR2 (*aiti-Mullerian hormone receptor, type II*) all involved in TGF-beta signaling pathway, ERBB4 (*erb - b2 receptor tyrosine kinase 4*) and ADRA1B (*adrenoceptor alpha 1B*) which are involved in the Calcium signaling pathway, FGFR1 (*fibroblast growth factor receptor 1*) and MAP3K12 (*mitogen-activated protein kinase kinase kinase 12*) which are involved in the MAPK signaling pathway, and THEM4 (*thioesterase superfamily member 4*) which is involved in the PI3K-Akt signaling pathway. In the LF group 68 fixed genome windows containing 113 candidate genes in the HF group were selected including, PROK1 (*prokineticin 1*), CHD7 (*Chromodomain helicase DNA binding protein 7*), KDM6A (*lysine K-specific demethylase 6A*), TENM1 (*teneurin transmembrane protein 1*), and SWI5 (*SWI5 homologous recombination repair protein*). In the shared window we found genes such as SYCP2 (*synaptonemal complex protein 2*), SOX5 (*SRY sex determining region Y - box 5*), and POU3F4 (*POU class3 homeobox4*). Figure 4 is an example of three types of selected genes KDM4 represents a unique candidate in the LF group (Fig. 4A), DNMT3B represents a unique candidate in the HF group (Fig. 4B), and SOX5 represents a shared candidate in both the HF and LF groups (Fig. 4C).

### Nonsynonymous and nonsense substitution in high and low fecundity goats

**Figure 5:**
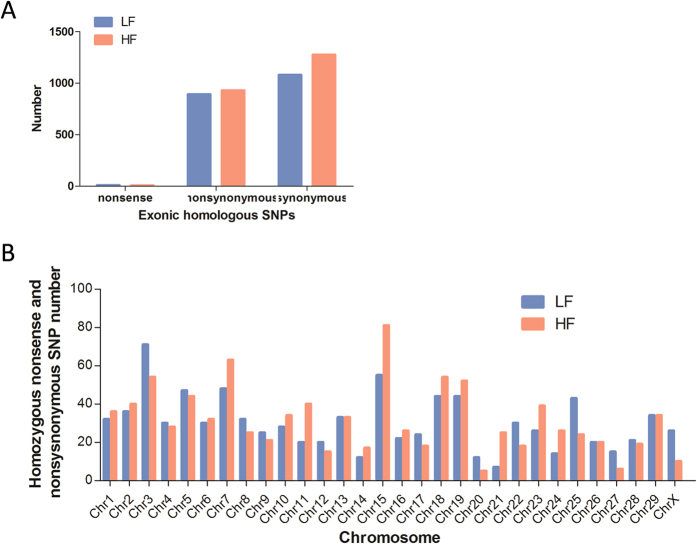
(**A**) Types of homologous called uniquely SNPs identified in the LF and HF groups. (**B**) The distribution of SNPs resulting in altered translation in each chromosome.

**Figure 6:**
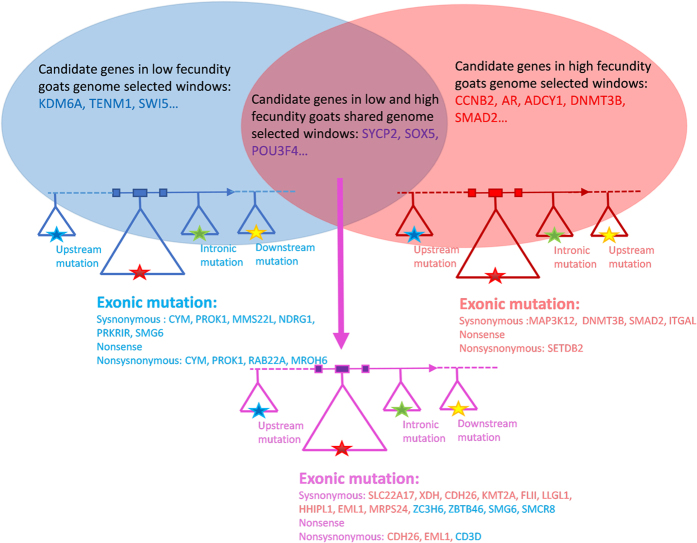
Summary of candidate genes and SNPs identified in each group.

In order to identify genetic markers useful for enhanced fecundity selection we screened the SNPs for nonsense and nonsynonymous homologous substitutions in both the HF and LF groups. Identification of these SNPs are important due to altering protein translation and potentially protein structure and function which may contribute to rapid evolution in domestic animals^19^,^20^. We found 931 nonsynonymous, 6 stop gain and 2 stop loss nonsense, and 1277 synonymous homologous SNPs specific to the HF group. Similarly, 893 nonsynonymous, 5 stop gain and 3 stop loss nonsense, and 1081 synonymous homologous SNPs, specific to the LF group, were identified (Fig. 5A). These results highlight that nonsynonymous nonsense substitutions may not play a predominant role in positive selection of fecundity traits. We determined the number and distribution of SNPs altering protein translation in the LF and HF groups and found that the number varied in chromosomes 7, 15, 21 and X (LF:HF = 48:63, 55:81, 7:25, 26:10) (Fig. 5B). Interestingly, in the SNPs resulting in altered translation only 7 colocalized with the HF unique candidate genes. SETDB2 (*SET domain, bifurcated 2*), LOC102187058 (*proteasome subunit beta type-6 pseudogene*) and LOC102191519 (*protocadherin gamma-C4*), colocalized with LF unique candidate genes CYM, PROK1, RAB22A (*RAB22A, member RAS oncogene family*) and MROH6 (*maestro heat-like repeat family member 6*), and 4 SNPs in the HF group colocalized in the shared candidate genes CDH26 (*cadherin 26*) and EML1 (*echinoderm microtubule associated protein like 1*), 1 SNPs in the LF groups colocalized in the shared candidate gene CD3D (*CD3d molecule, delta*) (Fig. 6; Table 5; Table S3).

**Figure 7:**
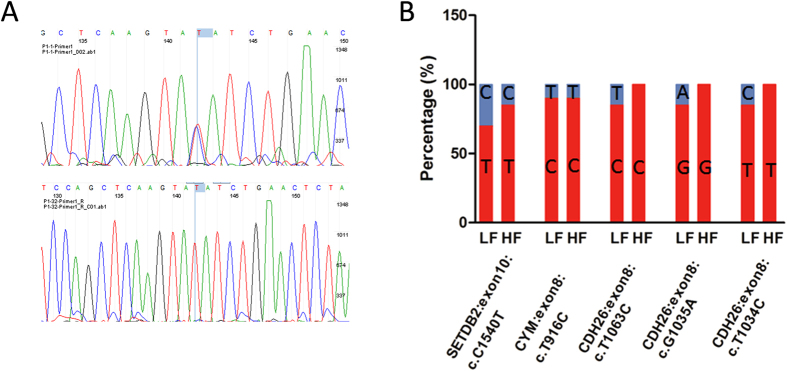
Validation of called altered translation SNPs. (**A**) Heterozygous and homologous exonic SNP localized in exon 10 (C1540T) in SETDB2 genes. (**B**) Percentages of 5 SNPs from random selected each of 10 samples from the LF and HF groups.

We then randomly selected 10 samples from the LF group and 10 samples from the HF group, and verified 5 SNPs by utilizing Sanger sequencing. 4 of the 5 SNPs showed the same trend in high throughput sequencing (C1540T in SETEB2, T1034C, G1035A, and T1063C in CDH26), while the exonic SNP in the CYM gene was not consistent with the high throughput sequencing (Fig. 7).

## Discussion

Our study provides a genome-wide differential scanning based on fecundity traits in dairy goats. After screening numerous substitutions were selected in the high fecundity population. To our knowledge this is the first selected sweep analysis study at the genome-wide scale completed in dairy goats.

Previous studies have demonstrated that selected sweep analysis can effectively help identify major and minor genes associated with domestic traits in many species. For example Rubin and his colleagues initially used selected sweep analysis and found that TSHR (thyroid stimulating hormone receptor) plays a pivotal role in the domestication process of chickens^21^. They have also demonstrated that *NR6A1*, *PLAG1*, and *LCORL* genes are associated with the elongation of the back through an increased number of vertebrae in domestic pigs^22^. Larkin using whole-genome sequencing with combined genotyping array methods demonstrated eleven candidate genes and ancestral SNPs related to milk-production, fertility, and disease resistance traits in bulls via artificial selection^23^.

In this study, we selected a naturally high fecundity Laoshan dairy goat population, with the view that some genetic substitutions may be naturally enriched or derived in genes associated with high fecundity. Using fixed window selection we screened the candidate genes associated with the high fecundity group. Identified examples included cyclin B2 which is encoded by CCNB2 and is known to activate Cdk1 in oocytes, a nullizygous mutation in cyclin B2 leads to smaller litter sizes in mice^24^,^25^. An androgen receptor which is responsible for transmitting androgen signals and when knocked out in female mice leads to a lower number of pups per litter^26^. The candidate genes ADCY1 encoding adenylate cyclase 1 is responsible for cyclizing AMP to form cAMP which takes part in oocyte meiotic arrest and resumption^27^. DNMT3B a member of the DNA methyltransferases (DNMTs) which appears to play a role in global DNA methylation and in human preimplantation embryo development^28^. SMAD2, a member of TGF-beta superfamily member that is responsible for oogenesis and some ovulatory processes which maintain normal female fertility^29^. AMHR was identified which is a negative factor in the regulation of ovulation, with dysfunction leading to anovulation in humans^30^. Heterozygous and homologous SNPs distributed in these locations or in the intergenic regions nearby may directly affect fecundity.

We particularly analyzed SNPs located in the exon region of these genes as they may play an important role in litter size determination. We counted the homologous SNPs that result in altered translation and were specifically found in the HF groups and identified only SETDB2. These results demonstrated that the substitution of exonic SNP c.C1540T was selected under artificial selection as well as altered the translation of SETDB2 in the HF group. SETDB2 is a histone methyltransferase, a previous study has demonstrated that after ovulation the expression of SETDB2 was down-regulated potentially playing a role in the early stages of luteinization^31^. We discovered that compared with the 928 nonsynonymous SNPs, 8 nonsense SNPs likely do not play primary roles in fecundity traits. Notably, nonsynonymous SNPs localized in the shared candidate genes, such as c. A65G in CD3D in the LF group, and c. A1063G, c. G1035A, c.T1034C in CDH26, and c.G560A in EML1 in the HF group. These nonsynonymous SNPs were selected as having strong genetic differentiation between the groups and may play important roles in fecundity.

The litter size trait which involving multiple genes and loci is complex quantitative trait. Besides, incorporating environmental factors such as husbandry, temperature and, precipitation intensity also turn the litter size into a malleable trait in many species^32^,^33^. Moreover, maternal effects have both environmental and genetic factors such as maternal age, intrauterine environment, milk production, and mothering ability may affect her offspring’s reproductive performance^34^. So, identification of the whole candidate genes and loci associated with prolificacy becomes a challenge of modern genetics. Due to the multiple contribution of genes in the complex quantitative litter size trait, previous studies concentrated on the association of one or few number genotype polymorphism(s) in reproductive related genes such as KISS, GDF9, POU1F1 and others may not lead to obvious improvement. High throughput screening for multiple genotype polymorphism combination become more and more important. However, due to the limitation of costing and experimental design, further verification of the newly found variations in large group is necessary.

In conclusion, we screened the SNPs found in dairy goats with different fecundity performance at a genome-wide scale. We identified candidate genes such as CCNB2, SYCP2, KDM6A and others that may take part in forming these traits. Furthermore nonsynonymous homologous SNPs in SETDB2, CYM, CDH26, EML1 and CD3D may play important roles in the fecundity traits seen in Laoshan dairy goats.

## Additional Information

**How to cite this article**: Lai, F.-N. *et al*. Whole-genome scanning for the litter size trait associated genes and SNPs under selection in dairy goat (*Capra hircus*). *Sci. Rep.* **6**, 38096; doi: 10.1038/srep38096 (2016).

**Publisher's note:** Springer Nature remains neutral with regard to jurisdictional claims in published maps and institutional affiliations.

## Supplementary Material

Supplementary Figures

Supplementary Tables

## Figures and Tables

**Table 1:** Primer information of Sanger sequencing.

Genes	Sequences (5′-3′)	Fragment size (bp)	Accession No.
*SETDB2*	F:GGTGCAGCCTATGGAGATTG	283	XM_005687460.2
	R:ACCACAGAATGAAACAAAGCCT		
*CYM*	F:GAGAAAGGAGGAGAGCTGGG	195	NM_001285759.1
	R:CACCCATGGCCCTTCTAAGA		
*CDH26*	F:ATGGGACTACATAAACCTA	680	XM_005688418.2
	R:CCAGTCACAGGGACGAGAT		

**Table 2:** Summary of sample information and sequencing quality.

Group	Low Fecundity	High Fecundity
Sample Size	20	14
Litter Size (Mean ± SD)	1.00 ± 0.00	3.07 ± 0.27
Raw Base (bp)	68,730,186,600	57,811,106,700
Clean Base (bp)	68,537,370,600	57,683,051,100
Effective Rate (%)	99.72	99.82
Error Rate (%)	0.04	0.05
Q20 (%)	94.74	95.2
Q30 (%)	87.87	87.31
GC Content (%)	45.07	44.21

**Table 3:** Summary of mapping information.

Group	Low Fecundity	High Fecundity
Mapped reads	449,856,898	486,550,427
Total reads	456,915,804	495,907,908
Mapping rate (%)	98.46	98.11
Average depth(X)	23.74	25.18
Coverage at least 1X (%)	99.51	99.52
Coverage at least 4X (%)	98.14	98.24

**Table 4:** Summary and annotation of SNPs.

Group		Low Fecundity	High Fecundity
Upstream		73,859	73,433
Exonic	Stop gain	440	380
	Stop loss	60	62
	Synonymous	43,697	43,330
	Non-synonymous	37,755	37,501
Intronic		3,519,630	3,513,651
Splicing		498	491
Downstream		70,317	70,199
Upstream/Downstream		922	908
Intergenic		8,697,962	8,669,931
ts		8,690,022	8,657,646
tv		3,768,689	3,765,482
ts/tv		2.305	2.299
Het rate (‰)		4.260	4.262
Total		12,458,711	12,423,128

**Table 5:** Altered translation SNPs colocalized in the HF group unique candidate windows.

Gene	Chromosome	Location	Amino acid			Type
			Ref	LF	HF	
SETDB2	Chr12	16931589	H	H/Y	Y	nonsynonymous
LOC102187058	Chr12	17045871	R	R/W	W	nonsynonymous
LOC102187058	Chr12	17046164	Y	Y/C	C	nonsynonymous
LOC102187058	Chr12	17046254	A	A/V	V	nonsynonymous
LOC102191519	Chr7	48613541	R	R/H	H	nonsynonymous
LOC102191519	Chr7	48617285	S		L	nonsynonymous
LOC102191519	Chr7	48671964	A		S	nonsynonymous
