# Supplementary material for: Whole-genome scanning for the litter size trait associated genes and SNPs under selection in dairy goat (Capra hircus)
Source: Sci Rep. 2016 Dec 1;6:38096. doi: 10.1038/srep38096 (PMC5131482; doi:10.1038/srep38096)
Supplement: Supplementary Figures [file srep38096-s1.pdf]

Supplementary Information

Whole-genome scanning for the fecundity traits under selection in dairy goat (*Capra hircus*)

Fang-Nong Lai [1], Hong-Li Zhai [2], Ming Cheng [3], Jun-Yu Ma [1], Shun-Feng Cheng [1], Wei Ge [1], Guo-Liang Zhang [1], Jun-Jie Wang [1], Rui-Qian Zhang [1], Xue Wang [4], Ling-Jiang Min [1], Jiuzhou Song [5], Wei Shen [1,*]

1 Key Laboratory of Animal Reproduction and Germplasm Enhancement in Universities of Shandong, College of Animal Science and Technology, Qingdao Agricultural University, Qingdao 266109, China;

2 Shandong International Biotechnology Park, Yantai 264670, China;

3 Qingdao Research Institute of Husbandry and Veterinary, Qingdao 266300, China;

4 College of Life Sciences, Qingdao Agricultural University, Qingdao 266109, China;

5 Department of Animal and Avian Sciences, University of Maryland, College Park, Maryland 20742, USA

Supplementary Figures

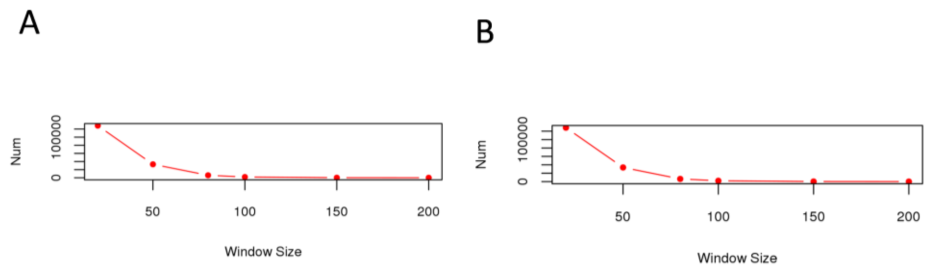

Figure S1. Line graph showing the counts of windows in which SNP numbers were < 20 in 50, 100, 150 and 200 KB window sizes in (A) the LF and (B) the HF groups.

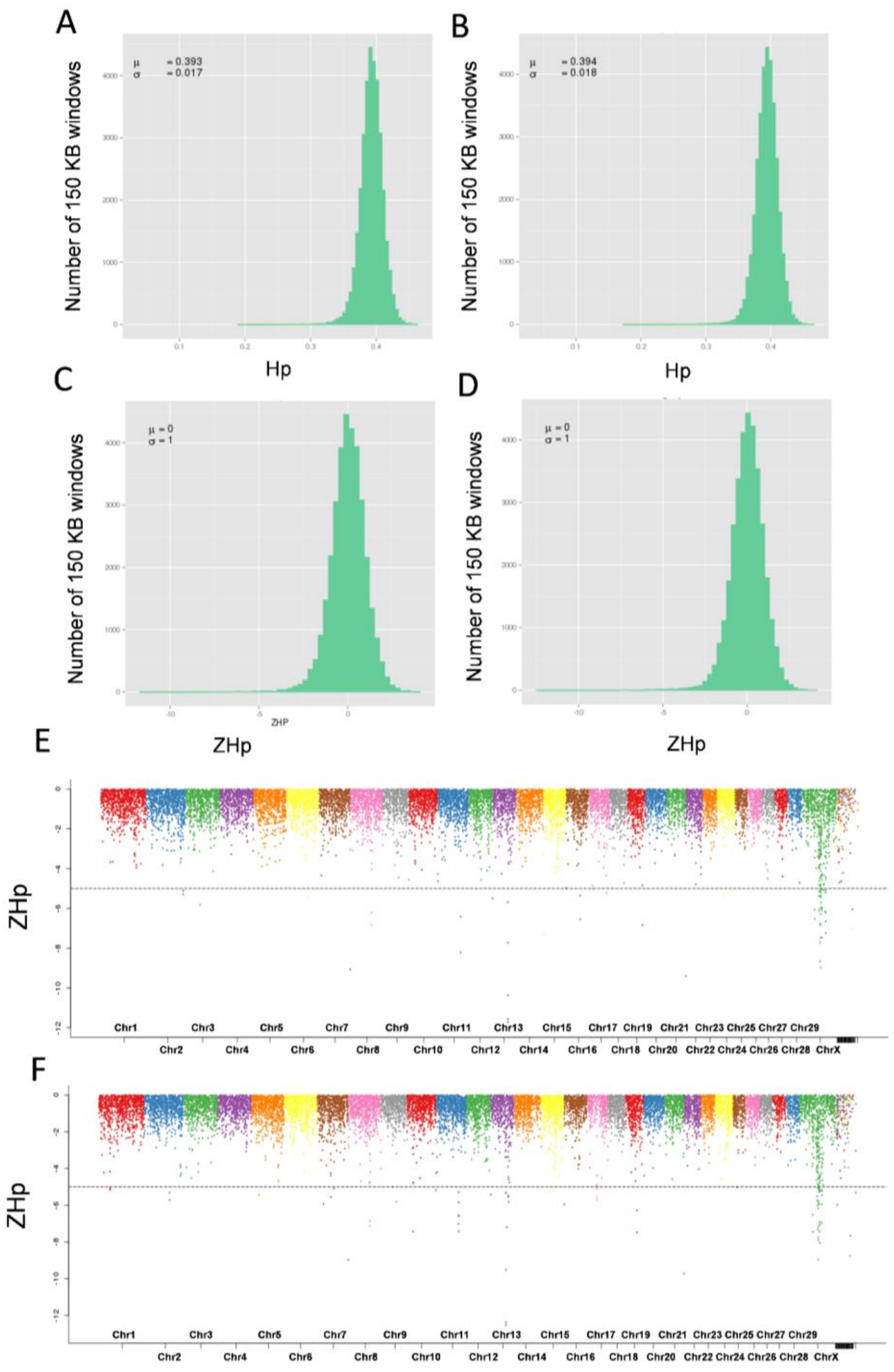

Figure S2. Histogram of Hp and ZHp for all 150 KB windows in the LF (A, C) and the HF (B, D) groups. Genome-wide ZHp Manhattan plot of the LF (E) and the HF (F) groups.

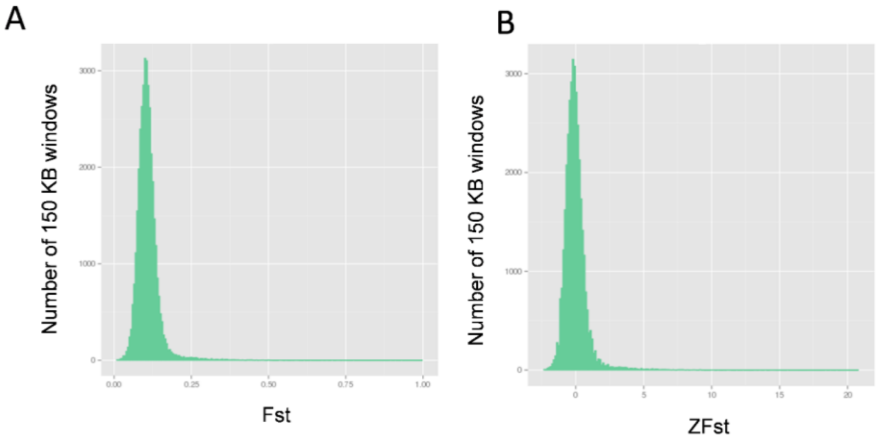

Figure S3. Histogram of Fst and ZFst for all 150 KB windows in the LF (A) and the HF (B) groups.
